# Sustained testosterone suppression: prognostic indicator in advanced hormone sensitive prostate cancer

**DOI:** 10.3389/fendo.2025.1652941

**Published:** 2025-10-13

**Authors:** Dongsheng Ma, Tao Zhuo, Xin Huang, Jianhong Xi

**Affiliations:** ^1^ Department of Reproductive Medicine, The Affiliated Bozhou Hospital of Anhui Medical University, Bozhou, China; ^2^ Department of Reproductive Medicine, The People’s Hospital Bozhou, Bozhou, China; ^3^ Department of Urology, The Fifth Affiliated Hospital of Xinjiang Medical University, Urumqi, China; ^4^ Department of Urology, Central Hospital of Chongqing University, Chongqing, China; ^5^ Department of Urology, The First Affiliated Hospital of Xinjiang Medical University, Urumqi, China

**Keywords:** testosterone suppression, castration, advanced hormone sensitive prostate cancer, androgen deprivation therapy, testosterone monitoring

## Abstract

**Objective:**

We aimed to investigate the relationship between sustained testosterone suppression and clinical outcomes in advanced hormone-sensitive prostate cancer (aHSPC), which integrates longitudinal testosterone with castration duration to predict tumor progression and prognosis.

**Methods:**

In this retrospective study, we analyzed 336 patients with aHSPC from two medical centers who underwent serial testosterone monitoring during androgen deprivation therapy (ADT). The patients were stratified by testosterone suppression sustainability into the testosterone sustained response and testosterone non-sustained response groups. We evaluated the baseline characteristics, time to progression (TTP), and the survival outcomes between groups.

**Results:**

The cohort demonstrated a median TTP of 18 months and an overall survival of 6.17 years. Patients in the testosterone sustained response group showed significantly better outcomes than those in the testosterone non-sustained response group, with longer median survival (7.58 *vs*. 3.00 years, *p*<0.001) and TTP (23.70 ± 14.66 *vs*. 13.68 ± 7.84 months, *p* < 0.001). Inverse correlations emerged between minimum testosterone and TTP (*r* = −0.238, *p* < 0.001) and between average testosterone and TTP (*r* = −0.220, *p* < 0.001). Multivariate analysis identified visceral metastases (adjusted OR = 0.45, 95%CI = 0.21–0.98, *p*=0.043) and high tumor load (adjusted OR = 0.53, 95%CI = 0.33–0.85, *p* = 0.008) as negative predictors of testosterone stabilization. The testosterone sustained response group status predicted reduced mortality risk (adjusted HR = 0.605, 95%CI = 0.369–0.990, *p* = 0.045), while higher minimum testosterone increased the mortality risk (adjusted HR = 1.358, 95%CI = 1.116–1.654, *p* = 0.002).

**Conclusion:**

Sustained testosterone suppression provides a clinically applicable method for assessing treatment efficacy and predicting prognosis in aHSPC.

## 1 Introduction

Prostate cancer (PCa) is a prevalent epithelial malignancy of the prostate, primarily affecting older men. With the trend of population aging and the widespread adoption of prostate-specific antigen (PSA) early screening, the prevalence and incidence of PCa have generally increased significantly worldwide (1–3). Notably, PCa ranks as the second most frequently diagnosed malignancy and the fifth leading cause of cancer-related mortality among men. While historically considered a low-incidence region compared to Europe and the United States, the incidence and mortality rates of PCa throughout Asia are increasing every year (4, 5). Established risk factors include advanced age, familial predisposition, and genetic susceptibility (6). PCa is an androgen-dependent malignancy in which androgen and the androgen receptor (AR) play a crucial role (7, 8), with the androgen/AR axis and the gonadotropin-releasing hormone (GnRH)/GnRH receptor pathway playing pivotal roles in the initiation and progression of PCa. Consequently, androgen deprivation therapy (ADT) remains the cornerstone of endocrine treatment for PCa, the aim of which is to induce and sustain castration level testosterone (T) suppression. Despite advances in novel hormonal therapies (NHTs) and targeted agents, ADT continues to serve as the backbone of combination regimens, administered continuously and long term in the majority of cases (9). Although ADT constitutes the cornerstone treatment for advanced hormone-sensitive prostate cancer (aHSPC), current clinical practice relies primarily on PSA monitoring to assess therapeutic efficacy while lacking systematic and standardized ongoing monitoring of serum testosterone, a more direct therapeutic target. Conventional endocrine therapy for PCa is typically satisfied by achieving traditional castration level (T < 50 ng/dl) or challenge castration level (T < 20 ng/dl) through single or intermittent testing, which fails to adequately address potential testosterone fluctuations and the phenomenon of “testosterone breakthrough” during treatment. This management approach, which prioritizes PSA monitoring over testosterone level, may lead to inadequate assessment of the efficacy of ADT and failure to detect potential treatment insufficiency in a timely manner. A critical focus of ADT is achieving profound and sustained testosterone suppression. Testosterone suppression sustainability is particularly relevant in aHSPC, where patterns of testosterone suppression stabilization may serve as prognostic indicators. Therefore, this study aimed to investigate the potential survival benefits for aHSPC associated with the depth (T < 20 ng/dl) and time (1 year) of sustained testosterone suppression through testosterone monitoring and validated using real-world clinical data from two Chinese clinical research centers.

## 2 Data and methods

### 2.1 Study population

From the follow-up database (*N*=1,917) of PCa patients in The Affiliated Bozhou Hospital of Anhui Medical University and the First Affiliated Hospital of Xinjiang Medical University, we identified 336 patients with aHSPC based on diagnostic, inclusion, and exclusion criteria.

### 2.2 Data collection and outcome measures

Clinical and demographic characteristics: age, smoking history, alcohol history, hypertension, and diabetes.

Tumor characteristics: initial PSA, clinical tumor stage (TNM classification), Gleason score, and tumor load.

Testosterone indicators: measured at multiple time points following ADT initiation, average testosterone, and minimum testosterone.

Continuity of ADT: continuous androgen deprivation therapy (CADT) and intermittent androgen deprivation therapy (IADT).

Tumor progression: time to progression (TTP) and TTP to metastatic castration-resistant prostate cancer (mCRPC).

Survival metrics: overall survival (OS), defined as the interval from PCa diagnosis to death or last follow-up.

ADT agents: All patients received luteinizing hormone-releasing hormone (LHRH) agonists (goserelin or leuprolide), with therapeutic equivalence confirmed through clinical validation.

Testosterone measurement: Serum testosterone was quantified using chemiluminescence immunoassay (CLIA). Both institutional laboratories maintained ISO15189 accreditation and certification from the China National Accreditation Service (CNAS).

### 2.3 Definitions used in the study

Advanced prostate cancer (aPCa): regionally or distantly metastatic PCa.

Advanced hormone-sensitive prostate cancer: metastatic PCa demonstrating therapeutic response to ADT.

Castration-resistant prostate cancer (CRPC): castration, serum testosterone T < 50 ng/dl, accompanied by biochemical (and) or imaging progression (10).

PCa progression: transition from aHSPC to CRPC, with TTP calculated from ADT initiation to CRPC diagnosis.

Testosterone stabilization without progression (TSP): sustained testosterone suppression, testosterone ≤20 ng/dl, sustained for >12 consecutive months.

### 2.4 Inclusion and exclusion criteria

The inclusion criteria were as follows: 1) newly diagnosed aHSPC meeting all conditions: no prior endocrine therapy and demonstrated an initial response to ADT; 2) treatment protocol adherence: CADT or IADT was initiated post-diagnosis; 3) monitoring compliance: three or more serum testosterone measurements during ≥12-month follow-up.

Patients were excluded based on the following criteria: 1) with other malignant tumors or combined severe cardiopulmonary diseases at baseline; 2) with incomplete clinical records; and 3) were lost to follow-up.

### 2.5 Sustained testosterone suppression

#### 2.5.1 Theory overview

Current clinical practice in ADT for PCa commonly relies solely on single or intermittent testosterone “testing” rather than “monitoring” to assess whether the testosterone levels have reached castration level, overlooking the testosterone fluctuations. Concurrently, “testosterone breakthrough” may occur during ADT due to androgen insensitivity; however, this is frequently neglected. The prognostic significance of testosterone for outcomes is frequently overshadowed by PSA levels (11), while advocates call for more appropriate testosterone monitoring (12) and a lower castration threshold (<20 ng/dl) (13, 14). ADT primarily suppresses testis-derived testosterone, but has minimal effects on the adrenal and tumor-derived androgens synthesized via paracrine/autocrine pathways. However, these residual very low levels of androgens are sufficient to activate the AR and drive cancer cell survival and proliferation (15, 16). Inter-individual variations in drug metabolism, adrenal androgen secretion capacity, and tumor-specific synthesis capabilities result in differing degrees of this “testosterone breakthrough” phenomenon. Given that residual androgens can drive the progression of PCa, combining ADT with agents that block androgen synthesis (e.g., abiraterone) or directly block androgens at the AR (e.g., enzalutamide, apalutamide, or darolutamide) (17–20) can achieve deeper and more complete sustained androgen suppression, thereby improving the survival outcomes of patients with aHSPC.

**Figure 1:**
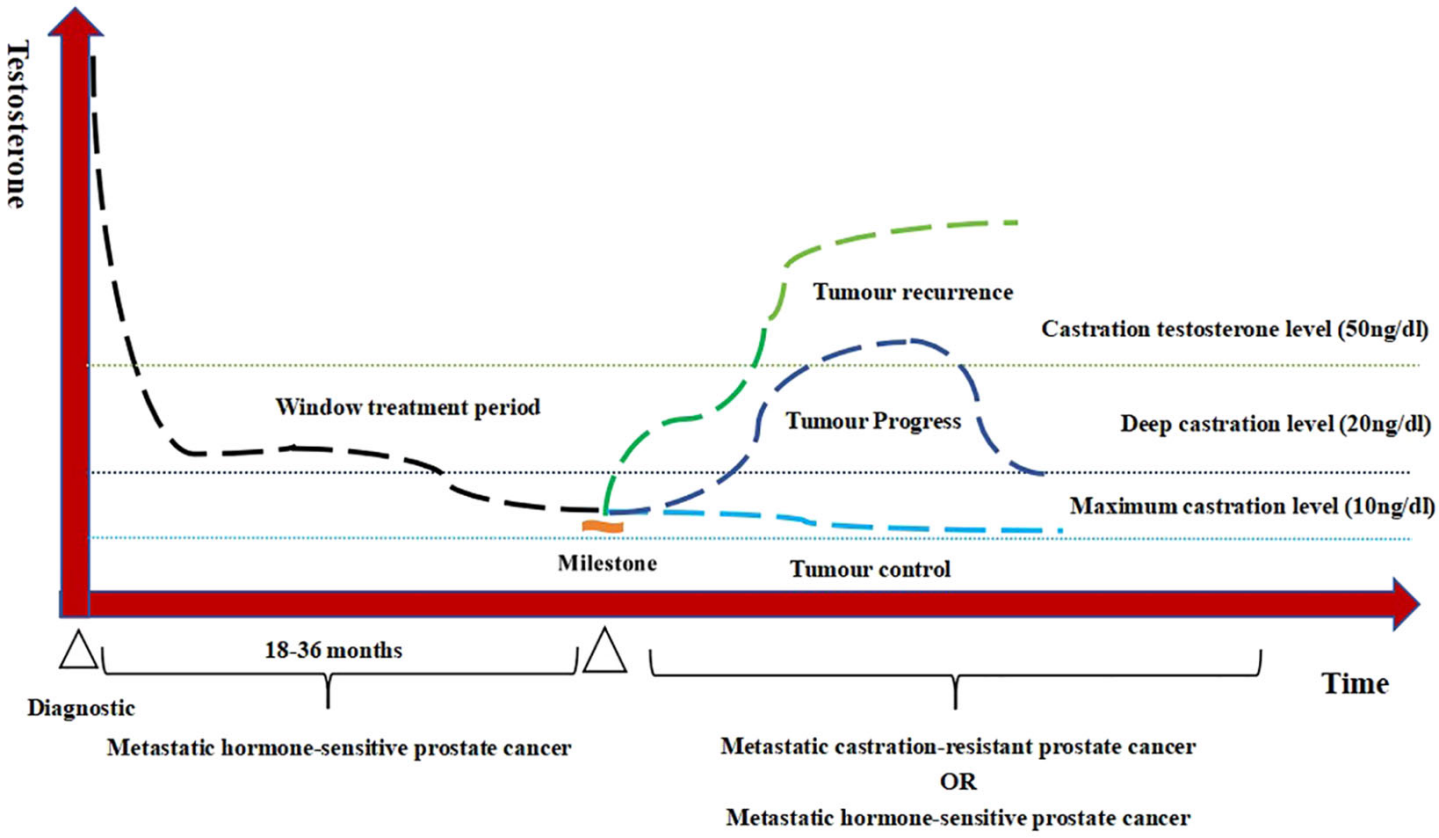
Testosterone patterns of androgen deprivation therapy (ADT) treatment in advanced prostate cancer.

Following ADT initiation, the testosterone levels in patients with PCa are maintained at castrate level, but exhibit temporal variations influenced by tumor progression dynamics and therapeutic interventions. These fluctuations create a quantifiable model when: time (*x*-axis) and serum testosterone levels (*y*-axis). This represents the first real-world applicable framework for quantifying the testosterone fluctuation patterns during ADT. This study moves beyond singular focus on non-rigorous and non-serial testosterone monitoring to an increased emphasis on testosterone monitoring and sustained testosterone suppression. **Figure 1** demonstrates the characteristic fluctuation patterns in advanced PCa. Testosterone is constantly changing over time and can be classified into different patterns such as continuous stability, fluctuating stability, and continuous progression (tumor control, tumor progress, and tumor recurrence).

**Figure 2:**
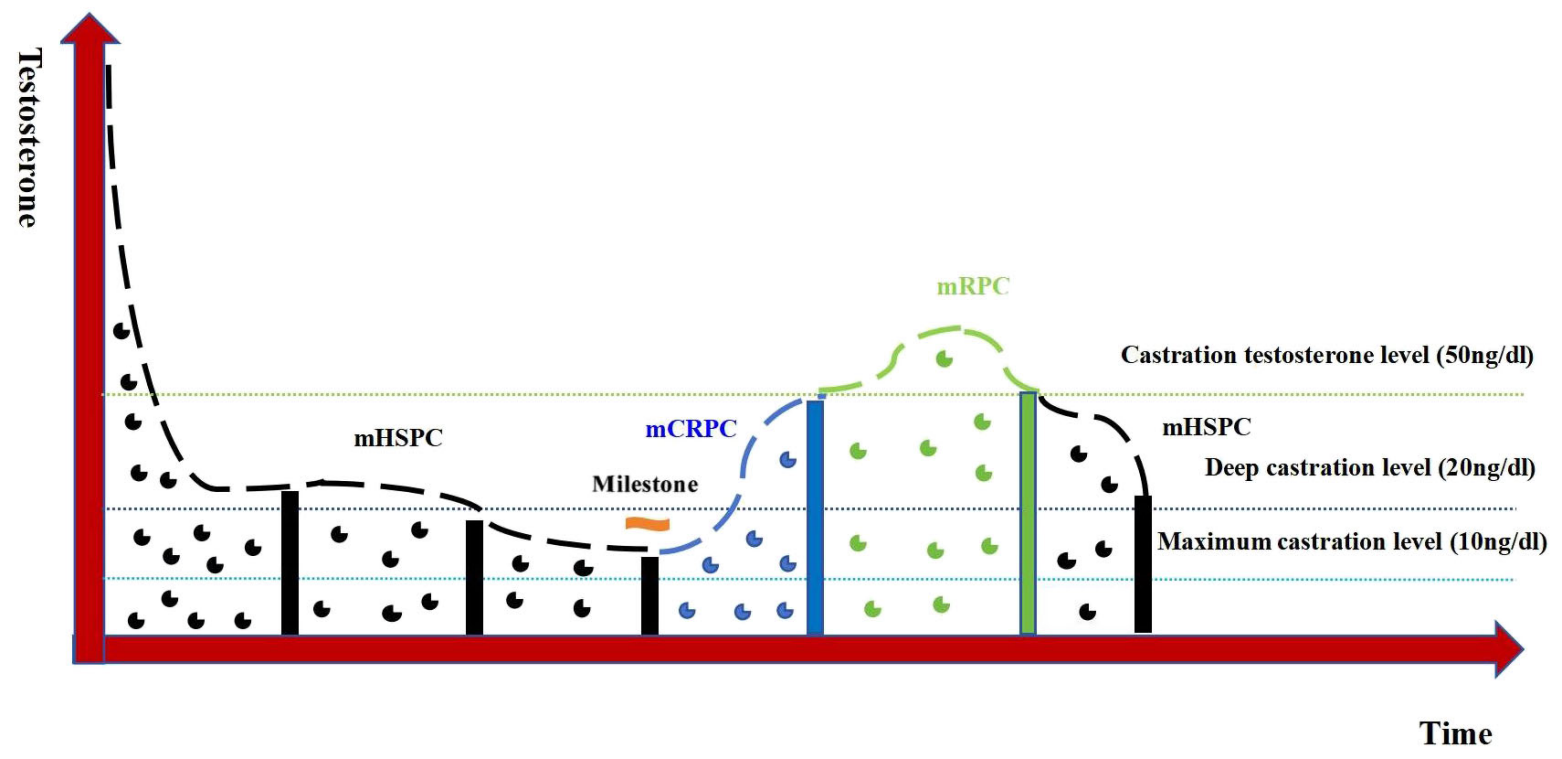
Testosterone patterns in advanced prostate cancer at different periods.

The treatment trajectory of PCa under ADT typically progresses through alternating phases of hormone sensitivity and castration resistance. Testosterone serves as a sensitive biomarker reflecting the evolution of PCa throughout these transitions. The testosterone patterns of advanced PCa at different periods are shown in **Figure 2**.

#### 2.5.2 Real-world significance of sustained testosterone suppression

In an ideal setting, if aHSPC received ADT with sustained testosterone suppression, this may predict tumor control and a better prognostic outcome might be achieved. Therefore, this study hypothesized that sustained testosterone suppression could be used as a prognostic indicator for survival assessment of aHSPC, which was validated by real-world data from two clinical research centers in China.

**Figure 3:**
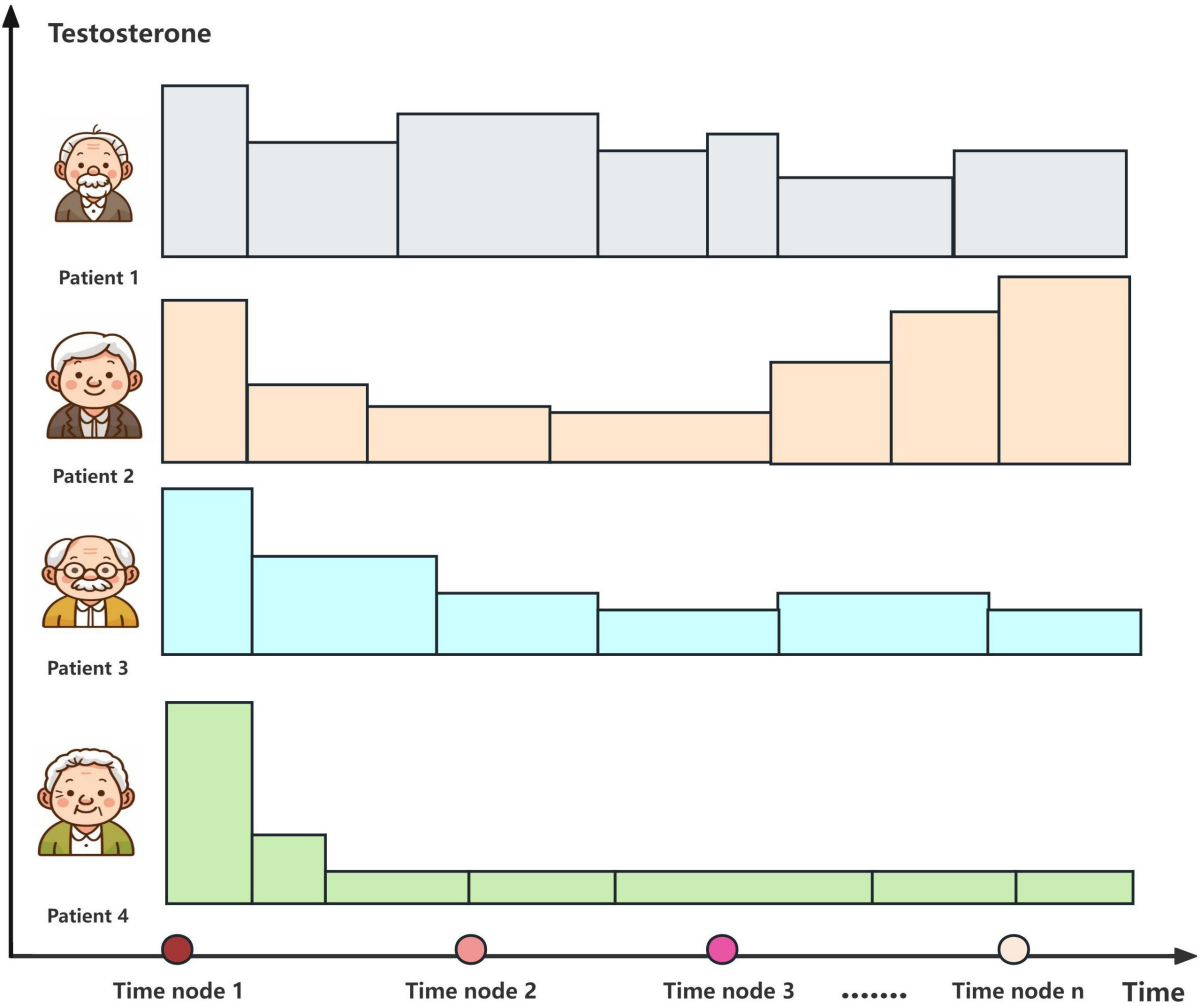
Testosterone suppression of androgen deprivation therapy (ADT) in patients with different types of advanced prostate cancer.

The study testosterone suppression time was set at 12 months of ADT, with the recommended castration testosterone level of 20 ng/dl. The testosterone sustained response group received sustained testosterone suppression (testosterone consistently <20 ng/dl), while the testosterone non-sustained response group had non-sustained testosterone suppression (any testosterone measurement >20 ng/dl) after initial suppression. **Figure 3** exhibits the testosterone suppression sustainability of the ADT trajectories across advanced PCa subtypes.

### 2.6 Ethical review

This study complied with the principles of the Declaration of Helsinki by examining the clinical information of retrospective patients. This study was approved by the Medical Ethics Committee of The People’s Hospital Bozhou (approval no. BYLS2024-147) and the Medical Ethics Committee of the First Affiliated Hospital of Xinjiang Medical University (approval no. K202504-46). The studies were conducted in accordance with local legislation and institutional requirements. The Ethics Committee Review Board waived the requirement for written informed consent for participation from the participants or the participants’ legal guardians/next of kin. The study did not involve patients’ personal privacy and disease characteristics, and coding anonymized identifiable information, consistent with waiving of informed consent.

### 2.7 Statistical methods

SPSS 26.0 and R 4.2.2 software were used for data processing and analysis. A *t*-test was used for comparisons between groups. Count data were described by rate, and the *χ* ^2^ test was used for comparisons between groups. Grade data were described by rate, and a rank-sum test was used for comparisons between groups. Survival time was calculated from the start of treatment after diagnosis, and death or still alive status (cutoff date May 2025) was considered as the end data, in months. Based on the results of the univariate analysis, variables with a *p*-value less than 0.05 were selected for inclusion into the multivariate analysis model. To reduce the impact of potential confounders, propensity score matching was employed. The test level for statistical analysis was *α* = 0.05.

## 3 Results

### 3.1 Baseline characteristics

The mean age was 72.04 ± 8.93 years in the overall group, and the median overall TTP was 18 months. There were no statistically significant differences in the age, diabetes, hypertension, smoking history, and alcoholism history variables between the two groups (*p* > 0.05), and the baseline characteristics were consistent. The overall median survival was 6.17 years, with a median survival of 7.58 years in the testosterone sustained response group and 3.00 years in the testosterone non-sustained response group. For the survival rates, the testosterone sustained response group had a significant survival advantage over the testosterone non-sustained response group (*p* < 0.001) (see **Table 1**).

**Table 1:** Kaplan–Meier estimates for survival rates (95%CI).

Characteristic	1 year	3 years	5 years	*P*
Overall	97.02% (95.22%–98.86%)	70.86% (65.27%–76.92%)	57.22% (50.17%–65.27%)	
Groups				<0.001***
Testosterone sustained response group	98.85% (97.58%–100.00%)	76.52% (70.70%–82.82%)	62.53% (54.61%–71.59%)	
Testosterone non-sustained response group	90.54% (84.11%–97.46%)	48.34% (35.74%–65.37%)	36.63% (24.21%–55.44%)	

*p < 0.05; **p < 0.01; ***p < 0.001 (same as below).

**Figure 4:**
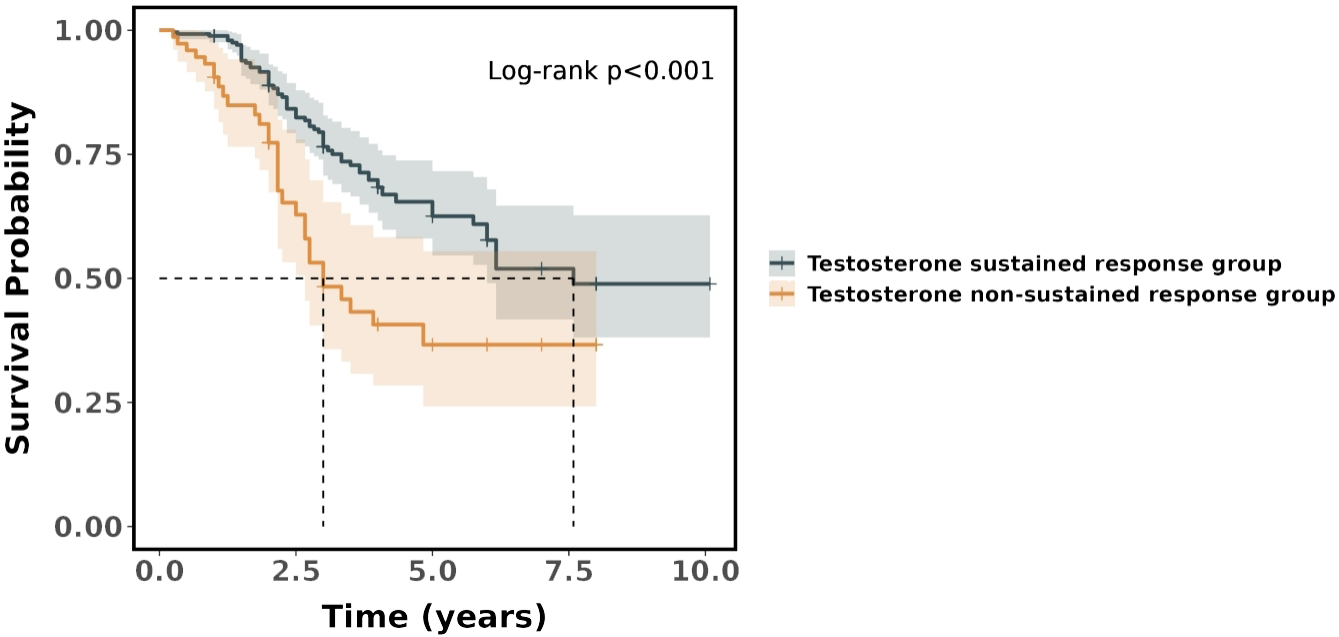
Kaplan–Meier survival curves for the two groups.

In the Cox regression model, it was found that the risk ratio (hazard ratio, HR) for the testosterone non-sustained response group was 2.21 (95%CI = 1.43–3.40), which indicated that the testosterone non-sustained response group had a 121% higher risk of death compared with the testosterone sustained response group. The difference in survival between the two groups was statistically significant (*p* < 0.001). The survival curves showed that the probability of survival was significantly higher in the testosterone sustained response group than that in the testosterone non-sustained response group (see **Figure 4**).

The multivariate Cox model for subgroup analysis was adjusted for baseline characteristics. In the subgroup analyses of tumor stage, tumor load, and Gleason score, there was no statistically significant difference between the testosterone sustained response and the testosterone non-sustained response group (*p* > 0.05). There was a significant difference between the testosterone sustained response and the testosterone non-sustained response group in the subgroup analysis of IADT and CADT (*p* = 0.014) (see **Table 2**).

**Table 2:** Subgroup analysis (multivariate Cox model).

Characteristic	Testosterone sustained response group	Testosterone non-sustained response group	Adjusted HR (95%CI)	*P* for interaction
Overall	67/262 (25.6)	30/74 (40.5)	2.19 (1.42–3.39)	
Continuity of ADT				0.014*
CADT	42/161 (26.1)	6/23 (26.1)	0.93 (0.39–2.20)	
IADT	25/101 (24.8)	24/51 (47.1)	2.88 (1.56–5.31)	
Tumor stage				0.365
4	38/125 (30.4)	14/33 (42.4)	2.07 (1.09–3.93)	
<4	29/137 (21.2)	16/41 (39.0)	3.41 (1.76–6.60)	
Tumor load				0.732
High	47/149 (31.5)	23/56 (41.1)	2.10 (1.26–3.50)	
Low	20/113 (17.7)	7/18 (38.9)	3.33 (1.30–8.53)	
Gleason score group				0.491
≥9	38/122 (31.1)	18/40 (45.0)	1.98 (1.09–3.58)	
<9	29/140 (20.7)	12/34 (35.3)	2.88 (1.39–5.99)	

*HR*, hazard ratio; *ADT*, androgen deprivation therapy; *CADT*, continuous androgen deprivation therapy; *IADT*, intermittent androgen deprivation therapy.

The baseline characteristics were balanced by propensity score matching in the testosterone sustained response group and testosterone non-sustained response group. A notable reduction in the standardized mean differences after matching can be observed, indicating that the propensity score matching effectively improved the balance of the covariates between the two groups, which performed 1:2 optimal pair matching. The TTP was significantly longer in the testosterone sustained response group compared with the testosterone non-sustained response group (23.70 ± 14.66 *vs*. 13.68 ± 7.84 months, *p* < 0.001) (see **Table 3**).

**Table 3:** Propensity score matching for time to progression (TTP).

Characteristic	Testosterone sustained response group (*n* = 148)	Testosterone non-sustained response group (*n* = 74)	*P*
TTP (months)	23.70 ± 14.66	13.68 ± 7.84	<0.001***

### 3.2 Correlation analysis of testosterone and tumor indicators

In the Pearson’s correlation analysis, the testosterone minimum and the testosterone minimum time (*r*=0.245, *p* < 0.001), the testosterone minimum and TTP (*r* = −0.238, *p* < 0.001), the average testosterone and the testosterone minimum time (*r*=0.228, *p* < 0.001), and the average testosterone and TTP (*r* = −0.220, *p* < 0.001), the above two parameters were weakly correlated with each other. There was a very high positive correlation between testosterone minimum and average testosterone (*r*=0.844, *p* < 0.001) (see **Table 4**).

**Table 4:** Correlation analysis results.

Parameter A	Parameter B	*R*	95%CI	*T*	*P*
Testosterone minimum	Average testosterone	0.844	0.810–0.872	28.78	<0.001***
Testosterone minimum	Initial testosterone	0.123	0.016–0.227	2.263	0.024*
Testosterone minimum	Testosterone minimum time	0.245	0.141–0.343	4.609	<0.001***
Testosterone minimum	TTP	−0.238	−0.337 to −0.135	−4.480	<0.001***
Testosterone minimum	Overall survival	−0.184	−0.285 to −0.078	−3.417	<0.001***
Average testosterone	Initial testosterone	0.161	0.055–0.264	2.987	0.003**
Average testosterone	Testosterone minimum time	0.228	0.124–0.327	4.276	<0.001***
Average testosterone	TTP	−0.220	−0.320 to −0.116	−4.122	<0.001***
Initial testosterone	Testosterone minimum	0.101	−0.006 to 0.206	1.853	0.065

*TTP*, time to progression.

### 3.3 Testosterone stabilization without progression univariate and multivariate analyses of influencing factors

The multivariate analysis revealed significant associations between several clinical characteristics and the testosterone stabilization without progression outcome. PCa patients with visceral metastasis had a lower likelihood of testosterone stabilization without progression outcome compared to those with no visceral metastasis (adjusted OR = 0.45, 95%CI = 0.21–0.98, *p* = 0.043). Similarly, a higher tumor load was associated with reduced odds of testosterone stabilization without progression outcome (adjusted OR = 0.53, 95%CI = 0.33–0.85, *p* = 0.008) (see **Table 5**).

**Table 5:** Univariate and multivariate analyses of the influencing factors (logistic regression).

Characteristic	Univariable					Multivariable				
	*N*	Event *N*	OR	95%CI	*P*	*N*	Event *N*	OR	95%CI	*P*
Age (years)	336	135	1.00	0.97–1.02	0.852					
Smoking history										
No	258	101	–	–						
Yes	78	34	1.20	0.72–2.01	0.483					
Alcoholism history										
No	308	126	–	–						
Yes	28	9	0.68	0.30–1.56	0.367					
Hypertension										
No	199	89	–	–		199	89	–	–	
Yes	137	46	0.62	0.40–0.98	0.041*	137	46	0.65	0.41–1.04	0.074
Diabetes										
No	249	107	–	–						
Yes	87	28	0.63	0.38–1.05	0.079					
Neurological invasion										
No	236	94	–	–						
Yes	100	41	1.05	0.65–1.69	0.842					
Visceral metastasis										
No	292	125	–	–		292	125	–	–	
Yes	44	10	0.39	0.19–0.83	0.014*	44	10	0.45	0.21–0.98	0.043^*^
Tumor stage										
<4	178	76	–	–						
4	158	59	0.80	0.52–1.24	0.318					
Gleason score group										
<9	174	76	–	–						
≥9	162	59	0.74	0.48–1.14	0.176					
Tumor load										
Low	131	66	–	–		131	66	–	–	
High	205	69	0.50	0.32–0.78	0.002**	205	69	0.53	0.33–0.85	0.008^**^
Initial PSA	336	135	1.00	1.00–1.00	0.180					
Initial testosterone	336	135	1.00	0.96–1.04	0.866					
Continuity of ADT										
IADT	152	46	–	–		152	46	–	–	
CADT	184	89	2.16	1.38–3.39	<0.001***	184	89	2.25	1.41–3.58	<0.001^***^

*PSA*, prostate-specific antigen; *ADT*, androgen deprivation therapy; *CADT*, continuous androgen deprivation therapy; *IADT*, intermittent androgen deprivation therapy.

### 3.4 Survival univariable–multifactor analysis

The multivariate analysis revealed several associations with mortality. Patients with diabetes exhibited a non-significantly higher risk of mortality compared to those without diabetes (adjusted HR = 1.406, 95%CI = 0.929–2.127, *p* = 0.107). In contrast, those with testosterone sustained response had a significantly lower mortality risk compared to those with non-sustained response (adjusted HR = 0.605, 95%CI = 0.369–0.990, *p* = 0.045). A higher minimum testosterone level was significantly associated with increased mortality (adjusted HR = 1.358, 95%CI = 1.116–1.654, *p* = 0.002), whereas average testosterone levels showed no significant association (adjusted HR = 0.910, 95%CI = 0.776–1.068, *p* = 0.248) (see **Table 6**).

**Table 6:** Univariate and multivariate analyses of the influencing factors (Cox regression).

Characteristic	Univariable					Multivariable				
	*N*	Event*N*	HR	95%CI	*P*	*N*	Event*N*	HR	95%CI	*P*
Age	336	97	1.012	0.989–1.036	0.297					
Smoking history										
No	258	62	–	–						
Yes	78	35	1.384	0.913–2.098	0.126					
Alcoholism history										
No	308	83	–	–						
Yes	28	14	1.511	0.857–2.664	0.154					
Hypertension										
No	199	49	–	–						
Yes	137	48	1.054	0.707–1.572	0.796					
Diabetes										
No	249	55	–	–		249	55	–	–	
Yes	87	42	1.680	1.123–2.515	0.012*	87	42	1.406	0.929–2.127	0.107
Neurological invasion										
No	236	73	–	–						
Yes	100	24	0.687	0.433–1.091	0.112					
Visceral metastasis										
No	292	80	–	–						
Yes	44	17	1.201	0.711–2.028	0.494					
Tumor stage										
<4	178	45	–	–		178	45	–	–	
4	158	52	1.599	1.070–2.390	0.022*	158	52	1.309	0.848–2.020	0.225
Gleason score group										
<9	174	41	–	–		174	41	–	–	
≥9	162	56	1.730	1.155–2.589	0.008**	162	56	1.484	0.971–2.268	0.068
Tumor load										
Low	131	27	–	–		131	27	–	–	
High	205	70	1.832	1.175–2.858	0.008**	205	70	1.352	0.841–2.174	0.213
Initial PSA	336	97	1.000	1.000–1.000	0.178					
Initial testosterone	336	97	0.982	0.945–1.020	0.338					
Continuity of ADT										
IADT	152	49	–	–						
CADT	184	48	0.683	0.458–1.017	0.060					
Testosterone suppression sustainability										
Testosterone non-sustained response	201	73	–	–		201	73	–	–	
Testosterone sustained response	135	24	0.480	0.302–0.762	0.002**	135	24	0.605	0.369–0.990	0.045^*^
Testosterone minimum time	336	97	1.045	0.991–1.102	0.106					
Testosterone minimum	336	97	1.256	1.163–1.356	<0.001***	336	97	1.358	1.116–1.654	0.002^**^
Average testosterone	336	97	1.153	1.076–1.235	<0.001***	336	97	0.910	0.776–1.068	0.248

*PSA*, prostate-specific antigen; *ADT*, androgen deprivation therapy; *CADT*, continuous androgen deprivation therapy; *IADT*, intermittent androgen deprivation therapy.

## 4 Discussion

The treatment paradigm for aHSPC has undergone a substantial evolution. While ADT remains the cornerstone treatment across disease stages, from localized to metastatic PCa, its role has expanded through combination strategies with novel agents. Pre-castration testosterone levels are associated with the risk of PCa development and progression, while post-castration testosterone levels are an important predictor of survival and prognosis in patients with PCa (21–24). The serum total testosterone levels in patients with PCa can gradually stabilize over time, the “time-dependency” theory (25). A prognostic indicator for survival assessment in aHSPC, sustained testosterone suppression, was proposed based on the treatment response to ADT. Sustained testosterone suppression represents the comprehensive integration of temporal testosterone patterns with therapeutic response, addressing a critical gap in prior research that focused exclusively on non-rigorous and non-serial testosterone monitoring while neglecting the crucial dimension of sustained testosterone suppression maintenance. By quantifying both the testosterone and duration of testosterone suppression, this standard provides a more physiologically relevant assessment of the efficacy of ADT in aHSPC.

The study cohort (mean age, 72.04 ± 8.93 years) demonstrated an overall median TTP of 18 months. The median OS was 6.17 years, with median survival of 7.58 years in the testosterone sustained response group and 3.00 years in the testosterone non-sustained response group. For the survival rates, the testosterone sustained response group had a significant survival advantage over the testosterone non-sustained response group (*p* < 0.001). In the Cox regression model, the risk ratio for the testosterone non-sustained response group was 2.21 (95%CI = 1.43–3.40), which indicated that the testosterone non-sustained response group had a 121% higher risk of death compared with the testosterone sustained response group. The duration of the first off-treatment interval (>40 weeks) was associated with a shorter time to CRPC (HR = 2.9, 95%CI = 1.1–7.7, *p* = 0.03) and death (HR = 3.8, 95%CI = 1.1–13.6, *p* = 0.04) (26). The comparative effectiveness analysis demonstrated comparable outcomes between IADT and CADT, locally advanced, or metastatic PCa patients who achieved initial therapeutic response. However, emerging evidence suggests the potential superiority of IADT in certain clinical contexts (27–30). The propensity score-matched cohorts balanced for demographic and clinical characteristics showed significantly prolonged TTP in the testosterone sustained response group (23.70 ± 14.66 months) compared with the non-sustained responders (13.68 ± 7.84 months, *p* < 0.001). Serum-free testosterone emerged as an independent prognostic factor for disease progression (HR = 0.93, 95%CI = 0.88–0.99, *p* = 0.029) (31), with IADT demonstrating a longer median time to CRPC than CADT (32). The correlation analyses revealed significant but weak inverse relationships between minimum testosterone and TTP (*r* = −0.238, *p* < 0.001) and between average testosterone and TTP (*r* = −0.220, *p* < 0.001), but demonstrated a strong positive correlation between minimum and average testosterone levels (*r*=0.844, *p* < 0.001). Optimal testosterone control during ADT (<20–30 ng/dl) significantly prolonged the therapeutic response in metastatic disease (33), although the prognostic value of maintaining testosterone <20 ng/dl was attenuated in patients with non-metastatic CRPC receiving first-line novel endocrine therapies (34). The definition of CRPC can be updated to “T < 20 ng/dl,” which may represent the true level of castration testosterone and may improve the treatment outcomes and prognosis of PCa (35).

The multivariate analysis identified visceral metastases, high tumor load, and ADT treatment interruptions as significant negative predictors of testosterone progression-free outcomes, with single visceral metastatic sites demonstrating superior survival compared with multiple visceral involvement (*p* < 0.01) (36–38). Tumor staging revealed a markedly worse prognosis for patients with mCRPC (M_1c_) compared to those with M_1a_ (lymph node metastasis) (*p* < 0.001) (39). The analysis confirmed a substantially elevated mortality risk in testosterone non-sustained responders (adjusted HR=0.605, 95%CI = 0.369–0.990, *p* = 0.045), with minimum testosterone levels showing a strong correlation with survival outcomes (adjusted HR= 1.358, 95%CI = 1.116–1.654, *p* = 0.002). Lower baseline serum testosterone was significantly associated with poorer survival outcomes in patients with aHSPC treated with CADT (40). Long-term follow-up of combined androgen blockade patients established 20 ng/dl as the critical testosterone threshold for optimal OS (*p* = 0.0048), emphasizing that sustained nadir achievement rather than a rapid testosterone decline serves as the principal prognostic determinant (41). The inherent variability of testosterone levels in clinical practice presents significant prognostic challenges, as a subset of patients with PCa fail to achieve target testosterone suppression even after 18–36 months of ADT. This observation raises critical questions about whether progressive upward trends and sustained fluctuations in the testosterone levels correlate with adverse clinical outcomes. Traditional non-rigorous and non-serial testosterone monitoring has provided limited prognostic value, whereas this study proposed that sustained testosterone suppression offers a more comprehensive, real-world evaluation of ADT. Notably, while some patients may achieve testosterone normalization within 12 months of ADT cessation, the recovery kinetics are significantly influenced by the pretreatment androgen status, with higher baseline levels predicting more rapid normalization (42).

**Figure 5:**
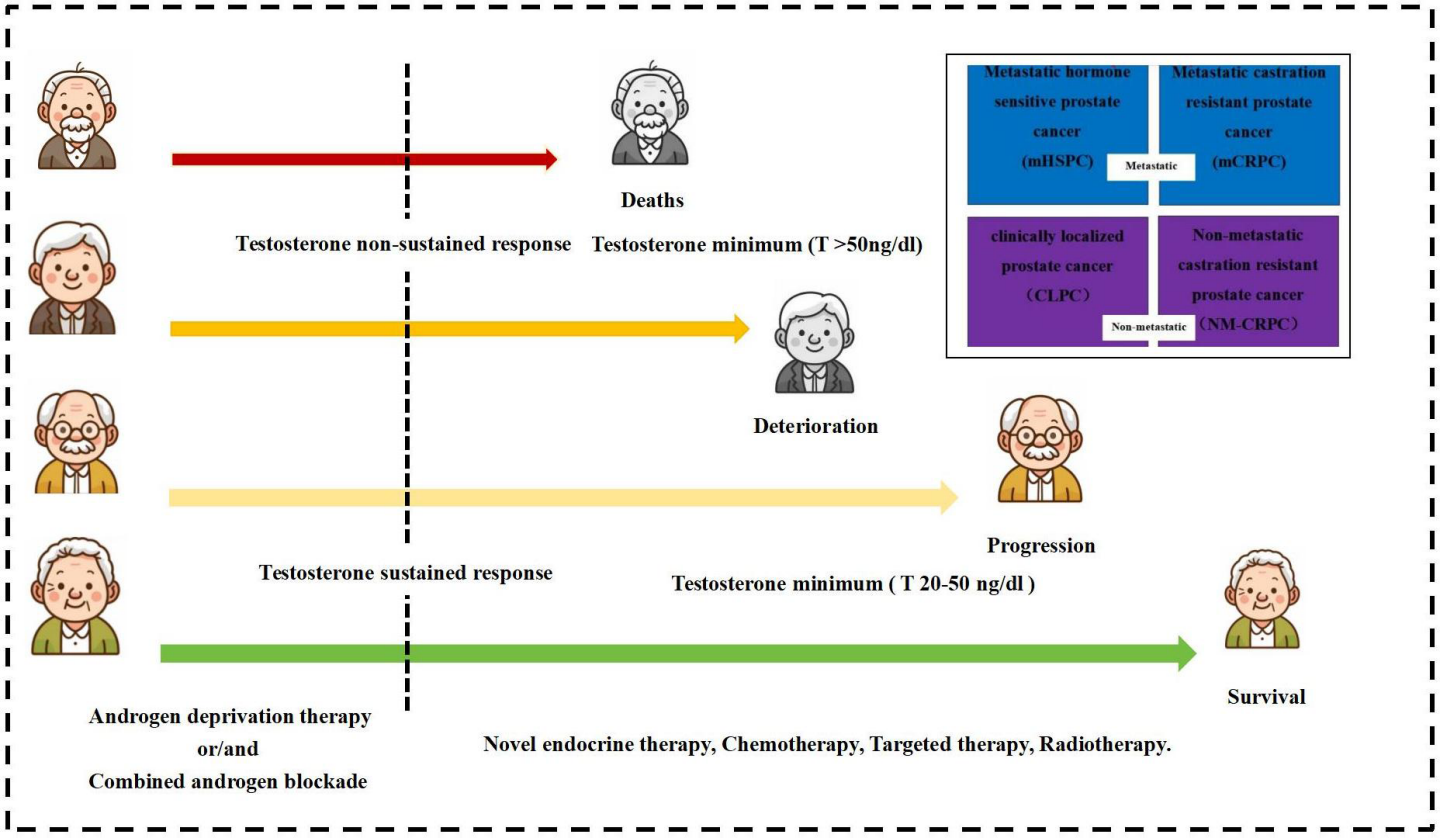
Testosterone patterns of death or survival in advanced prostate cancer.

The prognostic utility of serum testosterone monitoring during ADT management remains controversial despite extensive investigation of testosterone dynamics. In continuous testosterone monitoring, the serum testosterone levels and the “testosterone rebound” phenomenon can predict progression to CRPC (43). Serum testosterone levels may be considered an additional trigger for restarting treatment in IADT (44). ADT effectively controls cancer symptoms and extends survival, but induces testosterone deficiency associated with metabolic syndrome, insulin resistance, and hypogonadal symptoms, necessitating comprehensive monitoring of the hypothalamic–pituitary–testicular axis function in elderly patients (45). However, the clinical necessity of routine testosterone monitoring remains debated, particularly in the era of NHTs that have powerful testosterone suppression mechanisms of action and are capable of acting directly on the AR. Therefore, it is generally not necessary to rely on serum testosterone to determine whether a drug is working well. Current practice prioritizes the PSA kinetics and radiographic findings over subtle testosterone fluctuations when evaluating the efficacy of NHTs. **Figure 5** illustrates the distinct survival outcomes associated with different testosterone patterns during ADT in advanced PCa, highlighting the complex relationship between testosterone suppression and therapeutic response.

### 4.1 Clinical application scenarios and recommendations for sustained testosterone suppression

Sustained testosterone suppression represents a paradigm shift in assessing the efficacy of ADT by emphasizing the cumulative duration of castrate testosterone levels (<20 ng/dl) rather than relying solely on nadir values as a single indicator. This approach recognizes that, although sustained testosterone suppression suggests a response to ADT, long-term castration may paradoxically accelerate hypogonadism and compound metabolic toxicity. The clinical utility of the sustained testosterone suppression framework lies in its capacity to synergize with PSA kinetics for the early identification of patients at high risk, enabling timely therapeutic intensification. By quantifying the cumulative testosterone exposure, this standard provides an evidence-based approach to ADT optimization, particularly valuable for metastatic HSPC patients with low tumor load concerned about hypogonadal effects. Prognostically, sustained testosterone suppression for a long time (12 months) is associated with a good prognosis (5-year survival, >60%); on the other hand, no-sustained testosterone suppression for a long time suggests early drug resistance, rapid progression to metastatic castration resistance, and the need for intensive therapy or combination therapy. Persistent testosterone fluctuations may reflect residual PCa or adrenal compensatory androgens, serving as early warning signs of impending castration resistance. However, the hormonal regulatory mechanisms of PCa and their interactions with the metabolic microenvironment require further elucidation. In particular, the interactions between the androgen–thyroid hormone signaling pathways and the biological links between prostatic fat volume and ADT responsiveness warrant further investigation (46, 47). These mechanisms often occur in complex clinical settings characterized by multiple hormonal imbalances, metabolic abnormalities, and concurrent drug therapies. A deeper understanding of these interaction mechanisms and elucidation of the crosstalk between these pathways may provide a theoretical basis for the development of combined PCa targeting strategies or personalized treatments.

The sustained testosterone suppression represents advancement in PCa management by systematically characterizing the dynamic relationship between longitudinal testosterone fluctuations and tumor progression. Our findings demonstrate that sustained testosterone suppression provides critical insights into ADT response assessment and prognostic prediction in advanced PCa. Importantly, this approach facilitates timely initiation of combination therapies (triplet or quadruplet regimens) at optimal dosages to achieve rapid, profound, and sustained testosterone suppression—a strategy associated with improved survival outcomes. By transcending the limitations of non-rigorous and non-serial testosterone monitoring, sustained testosterone suppression offers a refined tool for personalized therapeutic decision-making in advanced PCa management.

### 4.2 Study highlights

This investigation presents three key contributions to the management of aHSPC: firstly, we introduced the concept of sustained testosterone suppression. This paradigm shift advances testosterone management strategies by integrating longitudinal hormonal patterns rather than relying on isolated measurements. Secondly, while grounded in physiological principles and validated through real-world data, the model acknowledges inherent limitations in clinical translation. Practical constraints including the monitoring frequency, the sample size restrictions, and the heterogeneous treatment regimens (incorporating chemotherapy, radiotherapy, and targeted immunotherapy) may affect generalizability. However, these real-world conditions precisely enhance the clinical relevance of the model by demonstrating utility in complex treatment environments using accessible monitoring techniques. Thirdly, sustained testosterone suppression addresses critical gaps in current practice by quantifying the relationship between sustained castration duration and testosterone fluctuations, parameters previously overlooked despite their prognostic significance for tumor progression. Sustained testosterone suppression during ADT reflects therapeutic efficacy and great tumoral outcomes. In summary, sustained testosterone suppression is an advanced concept beyond the traditional binary (yes/no to castration) and represents a more refined and dynamic management and evaluation paradigm in ADT treatment for PCa, which is closely associated with improved long-term prognosis of patients with aHSPC.

## Data Availability

The raw data supporting the conclusions of this article will be made available by the authors, without undue reservation.
